# Chemical Variations on the p53 Reactivation Theme

**DOI:** 10.3390/ph9020025

**Published:** 2016-05-13

**Authors:** Carlos J. A. Ribeiro, Cecília M. P. Rodrigues, Rui Moreira, Maria M. M. Santos

**Affiliations:** Research Institute for Medicines (iMed.ULisboa), Faculty of Pharmacy, Universidade de Lisboa, 1649-003 Lisboa, Portugal; cjacribeiro@ff.ulisboa.pt (C.J.A.R.); cmprodrigues@ff.ulisboa.pt (C.M.P.R.); rmoreira@ff.ulisboa.pt (R.M.)

**Keywords:** small molecules, MDM2 inhibitors, MDMX inhibitors, p53 activators, p53-MDM2 interaction inhibitors, p53-MDMX interaction inhibitors, mutant p53, wild-type p53

## Abstract

Among the tumor suppressor genes, p53 is one of the most studied. It is widely regarded as the “guardian of the genome”, playing a major role in carcinogenesis. In fact, direct inactivation of the *TP53* gene occurs in more than 50% of malignancies, and in tumors that retain wild-type p53 status, its function is usually inactivated by overexpression of negative regulators (e.g., MDM2 and MDMX). Hence, restoring p53 function in cancer cells represents a valuable anticancer approach. In this review, we will present an updated overview of the most relevant small molecules developed to restore p53 function in cancer cells through inhibition of the p53-MDMs interaction, or direct targeting of wild-type p53 or mutated p53. In addition, optimization approaches used for the development of small molecules that have entered clinical trials will be presented.

## 1. Introduction

The tumor suppressor p53 was discovered over 35 years ago and plays a pivotal role in the regulation of cell cycle, apoptosis, DNA repair, senescence and angiogenesis [1]. Its central role as tumor suppressor is undeniable, as observed by the increased predisposition to cancer in individuals with Li-Fraumeni syndrome, who inherit a mutant *TP53* gene, and in *Trp*-null mice [2]. Moreover, in virtually all cancers, loss of p53 function occurs, either directly due to the presence of a mutated form of *TP53*, or indirectly due to inactivation of the p53 signal transduction pathways. In tumors that retain wild-type p53 status (50% of all cancers), its function is usually inactivated by overexpression of endogenous negative regulators, such as murine double minute-2 (MDM2) and MDM4 (also known as MDMX) [3].

Cellular levels of the p53 protein are tightly regulated. In normal cells, and under physiological conditions, steady-state levels of p53 are maintained very low by its negative regulators, mainly MDM2 and MDM4. However, under cellular stress, such as DNA damage, hypoxia or oncogene activation, a range of differential posttranslational modifications of p53 are triggered that lead to p53 stabilization and activation, by promoting its release from repression and by inhibiting degradation. For instance, upon acute DNA damage, p53 stabilization is mostly achieved by phosphorylation mediated by upstream kinases such as ATM/ATR and/or CHK1/CHK2. Activated p53 binds to DNA and promotes the transcription of several target genes, culminating in a proper cellular response that is dictated by the nature of the stress, cell type and environment milieu. Under low levels of stress, p53 induces a transient G1 cell cycle arrest, while cells attempt to repair their genome. However, if the damage is too severe, activation of the p53 pathway results in cell death by apoptosis or senescence. By contrast, loss of p53 tumor suppressor activity allows the proliferation of cells that are damaged under stress conditions, potentially leading to uncontrolled proliferation that can result in tumor development [4,5,6].

Canonical p53 responses that lead to cell cycle arrest, senescence and apoptosis are extensively studied specially when triggered upon acute DNA damage. Recently, however, more attention is given to understanding p53 signaling in a tumor context, since distinct stresses and different responses that can facilitate/trigger tumor suppression have been described. These interesting p53 responses include inhibition of oncogenic metabolic reprogramming, activation of autophagy, communication endorsement within the tumor microenvironment, inhibition of stem cell self-renewal and reprogramming of differentiated cells into stem cells, and limiting invasion and metastasis [2,7].

## 2. Reactivation of p53 as a Therapeutic Strategy

It is well documented that the loss of p53 can induce tumor formation in mice, whereas its restoration often leads to a rapid regression of established *in situ* tumors [8], showcasing the anticancer therapeutic potential of p53 reactivation. Nevertheless, studies based on genetically engineered mice show an heterogeneous response to p53 restoration [9]. Furthermore, the key question for p53 reactivation strategy is whether or not this event will result in a selective effect on tumor cells as opposed to healthy tissues. It seems that a simple overexpression of p53 in cells is not sufficient to activate the p53 pathway. The restored p53 protein needs to be properly activated, and for that the transformed environment of tumor cells appears to be required [8,10]. For instance, studies using p53-MDM2 interaction inhibitors showed that in fact, in normal cells, the activation of p53 induces preferentially cell cycle arrest and not cell death, revealing therefore a more selective toxic effect on tumor cells [11,12]. The effect of p53 activation by this type of inhibitor in normal tissues has an immense interest from a therapeutic perspective due to the possibility of using it in monotherapy, as well as protector of normal cells in combination with more aggressive agents [11,12].

Throughout the last ten years, great advances were made in devising strategies to modulate p53, giving rise to several review papers on the subject [3,12,13,14,15,16,17,18,19,20,21,22,23,24,25]. Pharmacological p53 reactivation strategies for cancer therapy can be clustered in two major approaches based on p53 status. In tumors that retain wild-type p53 but have defects in p53 regulatory pathways, the main goal is to inhibit the function of negative regulators of p53 activation outcome. When p53 is mutated in tumors, the most common strategy consists in refolding the protein into a wild-type conformation to restore its function. In this review, emphasis will be given to small-molecules that restore p53 function in cancer cells. However, other strategies are also being pursued such as the use of peptides, stapled peptides and other oligomers to inhibit the p53-MDM2/X interactions [21], or the use of adenovirus-mediated p53 cancer gene therapy [26].

In this review, we will present an overview of the most relevant small molecules developed to activate p53. Table 1 presents all *in vitro* cell-free and cell-based methods used to determine the IC_50_ of the compounds discussed in this review, as well as the cell lines employed and their p53 status.

### 2.1. Targeting p53-MDM2 Interaction

Increased levels of p53 repressor MDM2 are present in many cancers, mainly through *MDM2* gene amplification or by activity loss of MDM2 inhibitor ARF. Therefore, targeting the p53-MDM2 interaction to reactivate p53 has emerged as a promising new cancer therapeutic strategy [11,27,28,29,30,31,32,33,34,35,36,37,38,39,40,41,42,43,44,45,46]. MDM2 and p53 regulate each other through an autoregulatory feedback loop [47]. Activation of p53 stimulates the transcription of MDM2, which in turn binds to the *N*-terminal transactivation domain of p53, disabling its transcriptional function. MDM2 also promotes the nuclear export of p53 and p53 proteasome-mediated degradation through its E3 ubiquitin ligase activity by promoting mono and polyubiquitination, respectively, at several lysine residues. These events result in decreased levels of p53 that will therefore reduce MDM2 expression, allowing p53 protein to potentially be activated again [45,48].

**Figure 1:**
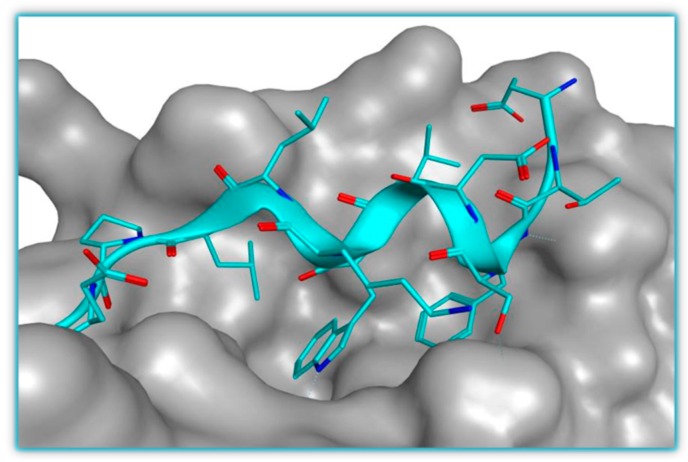
The p53-MDM2 interaction representation (PDB 1YCR). Phe19, Trp23 and Leu26 from a small amphipathic p53 derived α-helix (blue) are projected into the MDM2 pocket (grey surface).

The crystal structure of the p53 binding domain of MDM2 (109-residue amino-terminal) with a short peptide of the p53 transactivaction domain (15 residues) has been solved and published, providing detailed information about the interaction between these two proteins [49]. The co-crystal revealed that MDM2 has a deep hydrophobic cleft on which the p53 peptide binds as an amphipathic alpha helix. In the bound conformation, the p53 amphipathic α-residues 19-26 of the transactivation domain projects residues Phe19, Trp23 and Leu26 into the deep hydrophobic cleft of the MDM2 protein, representing the critical residues for binding between these two proteins to occur (Figure 1). In the crystal structure, Phe19 and Trp23 align in the deeper portion of the cleft. Phe19, through its backbone amine, forms one hydrogen bond with the backbone carbonyl Gln72 at the entrance of the cleft, while establishing hydrophobic interactions with Gly58 and Ile61 of MDM2. Trp23 occupies the deepest part of the binging pocket, forming a solvent protected hydrogen bond between the NH from its indole side chain and Leu54 of MDM2, and makes hydrophobic interactions with Gly58 and Ile61 of MDM2. Leu26 is the final residue of the alpha helix to be projected into the hydrophobic pocket. Furthermore, the interaction is strengthened by additional van der Waals contacts provided by p53 Leu22 [40,49].

After publication of the crystal structure of p53 bound to MDM2, several efforts were made to design more potent peptide derivatives and small molecules to target this interaction. Currently, several different chemical classes have been described as p53-MDM2 interaction inhibitors, and some small molecules are already in clinical trials [46,50].

#### p53-MDM2 Interaction Inhibitors

**Figure 2:**
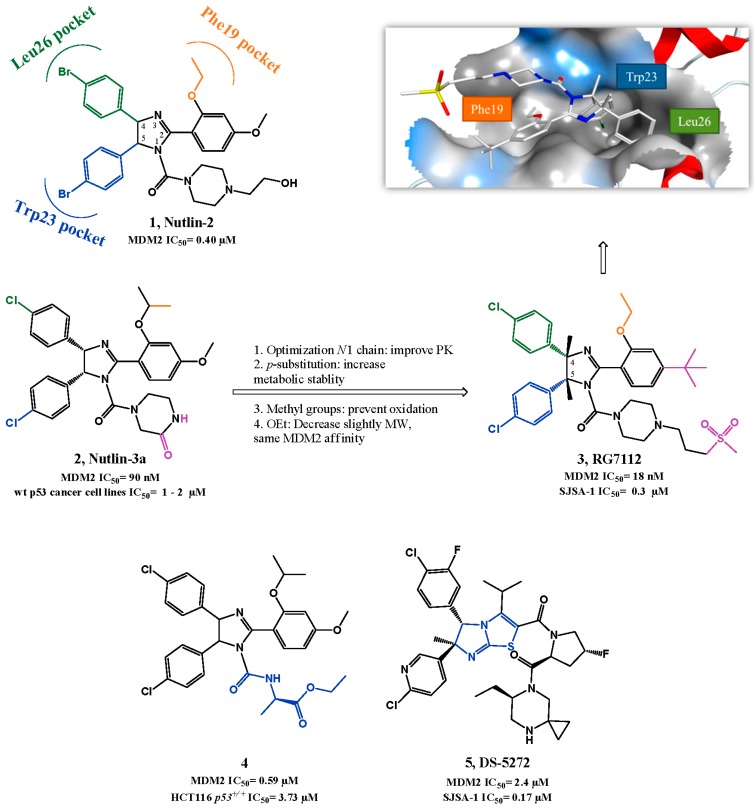
Nutlin scaffold optimization and examples of nutlin derivatizations. Right upper quadrant: crystal structure of compound **3** bound to MDM2 (PDB 4IPF). MDM2 surface is colored in blue for hydrophilic areas and grey for hydrophobic areas. Compound **3** is depicted in stick model and is colored according to element type: white for carbon atoms, blue for nitrogen atoms, red for oxygen atoms, yellow for the sulfur atom, and green for chlorine atoms.

During the last 15 years, several scaffolds have been described as p53-MDM2 interaction inhibitors and seven small molecules have advanced into clinical trials. The nutlin scaffold, consisting of a tetrasubstituted imidazoline unit, was first discovered by Hoffman-La Roche after a high-throughput screening (HTS) of a diverse library of synthetic compounds, using a surface plasmon resonance (SPR) assay, followed by structure-based optimization. This study provided three potent compounds for MDM2 and the first crystallographic structure of a small-molecule (nutlin-2: **1**, Figure 2) in complex with MDM2 [51]. In the crystallographic structure the *para*-bromophenyl ring at position 4 occupies Leu26*_(p53)_* pocket while the *para*-bromophenyl substituent at position 5 inserts deeply into the Trp23*_(p53)_* pocket with the bromo atom enhancing the binding by filling a small cavity not normally occupied by the indole ring of p53 Trp23. The Phe19*_(p53)_* pocket is occupied by the ethyl ether side chain of the third aromatic ring while its *para*-methoxy group mimics the p53 Leu22. The *N*1 chain functions mainly as a “solubility-tag” but also contributes to activity by possibly establishing polar interactions between the hydroxyl group and Gln72 side chain [51,52].

The most potent compound identified was the enantiopure nutlin-3a (**2**, SPR IC_50_ = 0.09 µM, MTT IC_50_ = 1–2 µM in wild-type p53 cancer cell lines), which has been used in monotherapy and in combination with other anti-cancer drugs and radiation, serving as proof-of-concept for nutlins and to establish p53-MDM2 interaction as a promising and valuable target [53,54,55,56,57,58]. However, the biological and pharmacokinetic (PK) properties of nutlin-3a were suboptimal for clinical development. The optimization of these properties was mainly focused on probing different *N*1 side chains to enhance PK properties and MDM2 binding and on removing stability liabilities found in the previous compounds (oxidation of the main core to imidazole, and metabolization of the *para*-methoxyphenyl group to phenol). The PK properties were amended by adding methyl groups to positions 4 and 5 of the imidazoline ring, and by replacing the methoxy with a *tert*-butyl group [59]. One of the best compounds, RG7112 (**3**, HTRF IC_50_ = 18 nM, MTT IC_50_ = 0.18–2.2 µM in wild-type p53 cancer cell lines) entered clinical trials [60]. RG7112 shows good selectivity over mutated p53 cancer cells (MTT IC_50_ = 5.7–20.3 µM), and it is able to activate the p53 signaling pathway in wild-type p53 cells, leading to cell cycle arrest and apoptosis. Furthermore a daily dose of 100 mg/kg is capable of promoting partly regression of SJSA-1 and MHM tumor xenograft mice models [46,61].

Hu *et al*. reported novel derivatives based on the imidazoline scaffold, mainly by varying the *N*1 side chain of nutlin-3. Compound **4** (FP IC_50_ = 0.59 µM, MTT HCT116 *p53*^+/+^ IC_50_ = 3.73 µM, Figure 2) was one of the most potent compounds obtained, although not representing an improvement of potency when compared with nutlin-3a. Nevertheless, these studies helped establishing that changing *N*1 side chain interferes mainly with PK properties but also with potency [62,63]. Several other analogs are disclosed in patents from Hoffman-La Roche, presenting the same imidazoline core and other structure variations such as imidazopyridinones [30,39,64,65]. Moreover, Miyazaki *et al*. also published a new series of dihydroimidazothiazole derivatives based on the nutlin-3a structure, such as DS-5272 (**5**, HTRF IC_50_ = 2.4 µM, LCVA SJSA-1 IC_50_ = 0.2 µM) [66,67].

The screening of a library of 338.000 compounds using a miniaturized affinity-based assay, termed ThermoFluor, identified the 1,4-benzodiazepine-2,5-dione scaffold (BDP) [68]. Selected compounds from this first screen were further tested employing a fluorescence polarization (FP) assay to detect specific p53-MDM2 interaction inhibitors.

**Figure 3:**
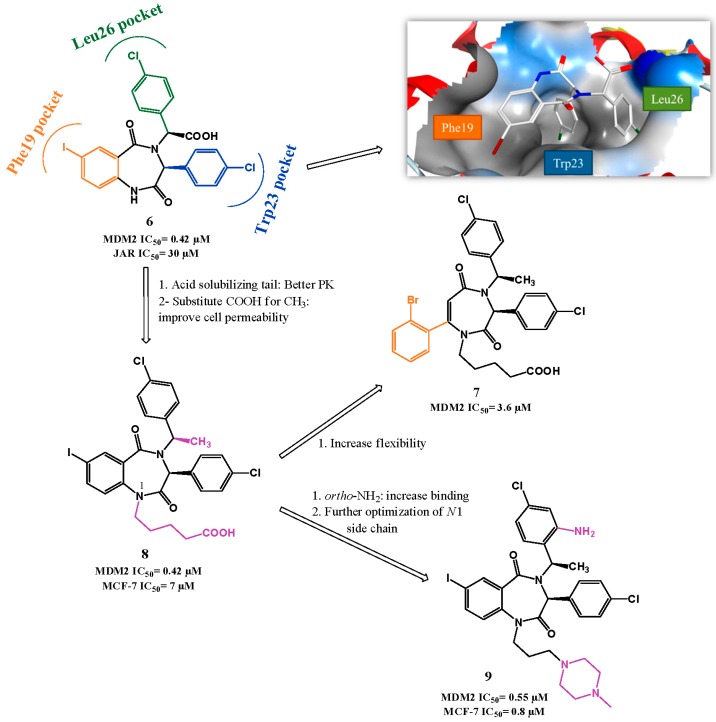
Benzodiazepinediones scaffold optimization. Right upper quadrant: crystal structure of compound **6** bound to MDM2 (PDB 1T4E). MDM2 surface is colored in blue for hydrophilic areas and grey for hydrophobic areas. Compound **6** is depicted in stick model and is colored according to element type: white for carbon atoms, blue for nitrogen atoms, red for oxygen atoms, dark red for the iodine atom, and green for chlorine atoms.

The confirmation of this class of compounds as feasible MDM2 inhibitors evoked a more detailed study in which a library of 22.000 BDP compounds was synthetized and screened using the two methods described above [69]. In 2005, a first SAR study by Johnson & Johnson gave rise to the lead compound **6** (FP IC_50_ of 0.42 µM, BrdU JAR IC_50_ = 30 µM, Figure 3).

The BDP:MDM2 co-crystal structure elucidated the interaction: 1,4-diazepine core gives the necessary rigidity from which the two chlorophenyl groups mimic perfectly Leu26 and Trp23 of p53, while the 7-iodophenyl group inserts in the Phe19*_(p53)_* pocket [68,70]. Although this last group does not insert as deeply as p53 Phe19 in the pocket, it was later rationalized that this interaction is enhanced because iodine atom makes contacts to the carbonyl group of backbone Gln72 with a strength comparable to a weak hydrogen bond [71]. The initial observation that BDP iodophenyl and p53 Phe19 were not superimposable, gave rise to a rational design of a novel 1,4-diazepine scaffold. In this new scaffold, an increased flexibility was introduced to the fused phenyl-diazepine rings in an attempt to ameliorate the Phe19 mimetic effect, while maintaining the orientation of the two chlorophenyl groups. Unfortunately, although this approach produced new active compounds, the FP IC_50_ values attained were higher in comparison to the original series (best compound: **7**, FP IC_50_ of 3.6 µM) [72].

Due to the poor PK properties of compound **6**, modifications were made to try to improve solubility and permeability. It was rationalized that the inclusion of substituents in *N*1 might be tolerated since it is primarily solvent-exposed in the co-crystal structure, and also changing the carboxylic acid could convey better PK properties to the scaffold. Several solubilizing groups were inserted to *N*1 and ultimately the pentanoic acid group was selected for further PK optimization. In this study, it was found that the acid group was important to activity, possibly by establishing a hydrogen bond to MDM2 Ser17, and most importantly by placing the chlorophenyl group in the correct orientation through steric repulsion. This repulsion orientation was maintained when carboxylate was substituted with methyl group, while increasing cell permeability (**8**, FP IC_50_ = 0.70 µM, BrdU MCF-7 IC_50_ = 7 µM) [73].

Searching for more potent BDP led to compound **9** bearing an *ortho* amino group in the *N*-benzylic ring (FP IC_50_ = 0.55 µM, BrdU MCF7 IC_50_ = 0.8 µM) responsible for an additional hydrogen bond established with the carbonyl of MDM2 Val93 [74,75]. Compound **9** was found later to have a synergistic outcome in association with doxorubicin, allowing the visualization of doxorubicin-mediated *in vivo* activity in a xenograft model at doses that are inactive in monotherapy treatment [76].

**Figure 4:**
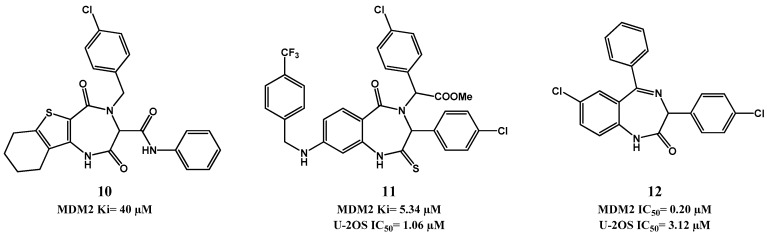
Examples of benzodiazepinedione derivatizations.

More recently, two new scaffolds based on the principle of bioisosterism of BDP have been reported: 1,4 thienodiazepine-2,5-diones (TDZ) [77] and thiobenzodiazepines (Figure 4) [78,79]. For TDZ only a cell-free binding screening has been reported, from which compound **10** emerged as lead compound with a FP *K*i of 40 µM [77]. The synthesis and biological evaluation of thiobenzo-diazepines showed that the simple replacement of the oxygen by a sulfur atom increased the potency in a FP binding assay, but not in cell-based evaluation. In this SAR study compound **11** emerged as a potential lead compound for future optimization with a FP *K*i of 5.34 µM and MTT U-2OS IC_50_ of 1.06 µM [78]. Continuation of this work resulted in compounds with better affinity to MDM2, but without cell-based assay improvement [79]. More recently, new benzodiazepine analogues were reported, but again without showing potency improvement (the best derivative, **12**, has FP *K*i = 0.2 µM, MTT U-2OS IC_50_ = 3.12 µM) [80]. Furthermore, these new scaffold derivatives did not show selectivity toward cells with wild-type p53 as observed for 1,4-benzodiazepine-2,5-dione derivatives (e.g., compound **9** is 10 times more selective, MCF-7 *vs.* MDA-MB-231 [75]).

**Figure 5:**
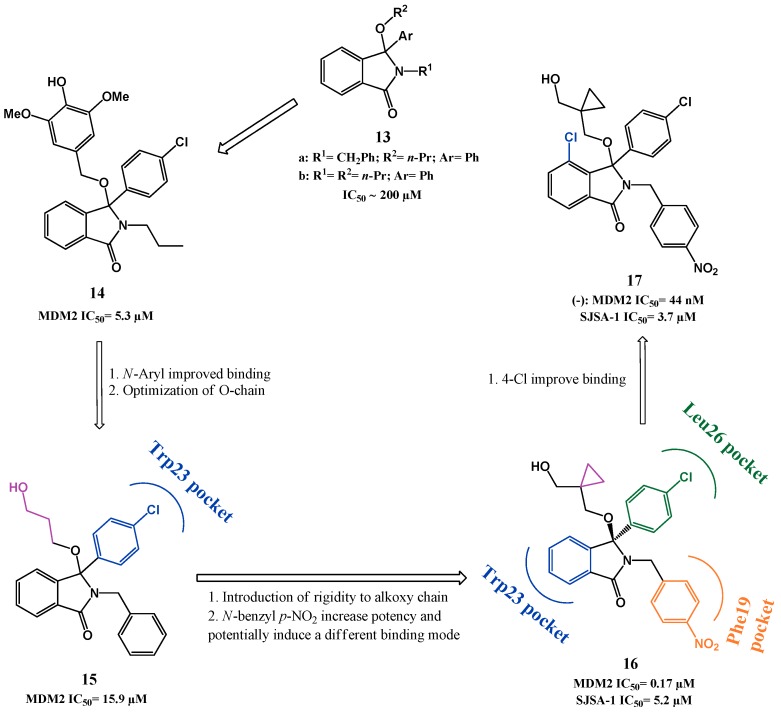
Isoindolinone scaffold optimization. Representations of binding modes were determined from chemical shift data and molecular docking [83,85].

Hardcastle *et al.* described inhibitors of the p53-MDM2 interaction based on an isoindolinone scaffold. Compounds bearing this template (**13a**,**b**, Figure 5) were first identified as modest inhibitors of the p53-MDM2 interaction (IC_50_ ~200 µM) in an *in vitro* p53-MDM2 binding assay screening study. In a first optimization, a small compound library focused on the isoindolinone core was synthesized guided by *in silico* ligand-design, using the published MDM2 crystal structure. Compound **14** emerged as a lead compound with an IC_50_ of 5.3 µM in a cell-free ELISA binding assay. In addition, compound **14** induced a dose-dependent increase of p53 transcriptional activity in the SJSA-1 cancer cell line [81,82]. In this first study, it was suggested that the introduction of different substituents into the isoindolinone template allowed different orientations of the inhibitors in the hydrophobic MDM2 pocket consequently making SAR studies more difficult to interpret. This statement was later corroborated by NMR experiments in which four different binding modes in twelve isoindolinones analyzed were identified, differing only in one group attached to the isoindolinone scaffold [83].

Considering the different binding modes and structure information gained by the NMR experiments, compound **15** (ELISA IC_50_ = 15.9 µM) was selected as lead compound for further optimization. The binding mode model of this compound suggested that introducing rigidity to the alkoxy side chain and adding substituents to the *N*-benzyl moiety could favor interaction with MDM2, giving rise to compound **16** (ELISA IC_50_ = 0.17 µM, SRB SJSA-1 IC_50_ = 5.2 µM) [84,85]. In the last study published by this group attempts to increase potency were made through modifications in the aromatic ring of the isoindolinone core, revealing that the introduction of a 4-chloro in isoindolinone ring improved binding (**17**(−), IC_50_ = 0.044 µM, SRB SJSA-1 IC_50_ = 3.7 µM) [86]. Furthermore contrary to compound **16**, compound **17** showed selectivity (3- to 4-fold) towards cells with wild-type p53.

**Figure 6:**
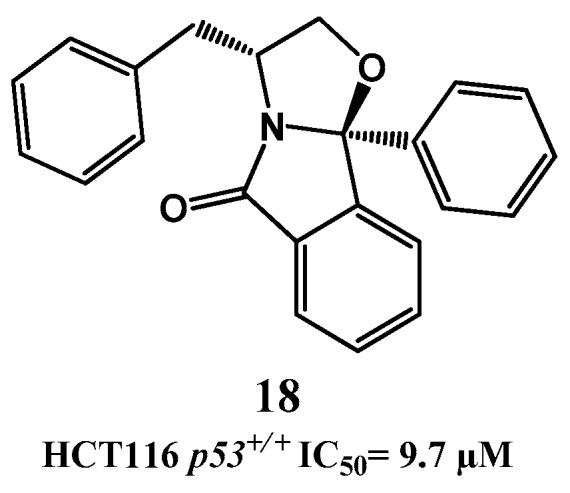
Oxazoloisoindolinone derivative **18**.

Recently, phenylalaninol-derived oxazoloisoindolinone **18**, containing a more rigid structure, was identified as a potential p53-MDM2 interaction inhibitor inducing a p53-dependent activation *in vitro* leading to apoptosis (SRB HCT116 *p53*^+/+^ IC_50_ = 9.7 µM, Figure 6) [87].

**Figure 7:**
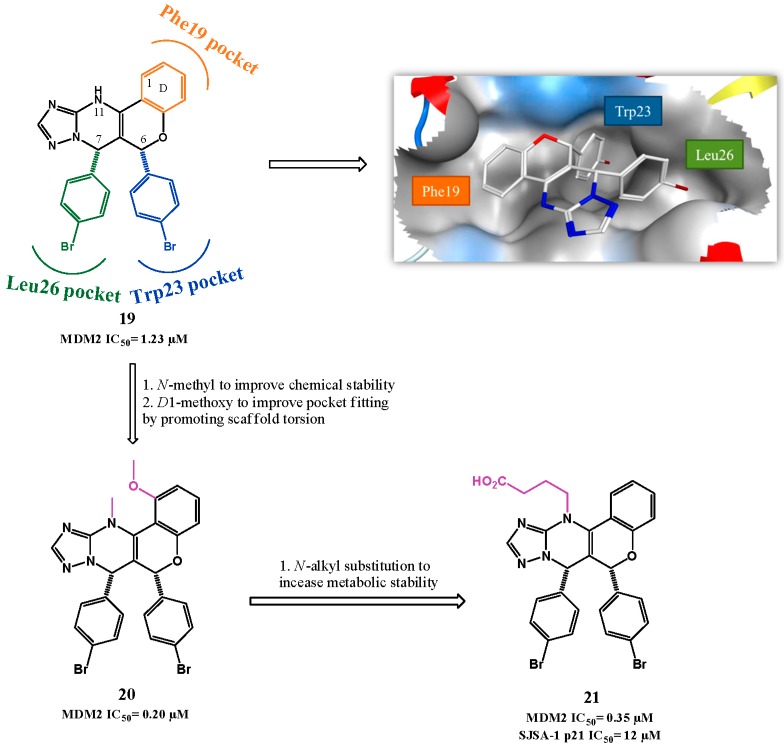
Chromenotriazolopyrimidine scaffold optimization. Right upper quadrant: crystal structure of compound **19** bound to MDM2 (PDB 3JZK). MDM2 surface is colored in blue for hydrophilic areas and grey for hydrophobic areas. Compound **19** is depicted as a stick model and is colored according to element type: white for carbon atoms, blue for the nitrogen atom, red for the oxygen atom, and dark red for bromine atoms.

Chromenotriazolopyrimidines were found by Amgen to be inhibitors of p53-MDM2 in a HTRF-based high throughput screen of about 1.4 million compounds, and their binding to MDM2 was confirmed by SPR. From this screening hit compound **19** (Figure 7) emerged [88]. Only the *syn*-(6*R*,7*S*) isomer was found to be active (HTRF IC_50_ = 1.23 μM). Co-crystallization of **19** with MDM2 showed that the chromenotriazolopyrimidine is a fairly rigid scaffold from which two *para*-bromophenyl groups at C-6 and C-7 in a *syn* relationship interact with MDM2 in the Trp23*_(p53)_* and Leu26*_(p53)_* pockets, respectively. Furthermore the C-7 aromatic group also establishes a weak π-stacking interaction with the His96 side chain of MDM2. The third key p53 amino acid Phe19 is mimicked by the chromene D ring. However, these compounds were chemically unstable and individual diastereoisomers tended to equilibrate, forming mixtures consisting mostly of the more stable *anti* diastereoisomers. This problem was overcome by *N*11-methylation, which prevented racemization without affecting the potency. Optimization of the lead compound was attempted by introducing variability to the three phenyl rings involved in the binding. Introduction of a methoxy group in position 1 gave the best cell-free activity (**20**, HTRF IC_50_ = 0.20 μM). However, it is believed that this improved activity is product of the molecule core torsion due to *N*-methyl and C1-methoxy proximity that allows a better pocket fitting rather than a improve interaction to the protein by the methoxy group [88].

To enhance PK properties, especially problems observed with metabolic stability hypothesized due to rapid *N*-demethylation and consequent tautomeric isomerization, SAR studies were performed at the *N*-substituent. It led to compound **21** (HTRF IC_50_ = 0.35 μM, SJSA-1 p21 IC_50_ = 12 μM), with increase metabolic stability [89].

**Figure 8:**
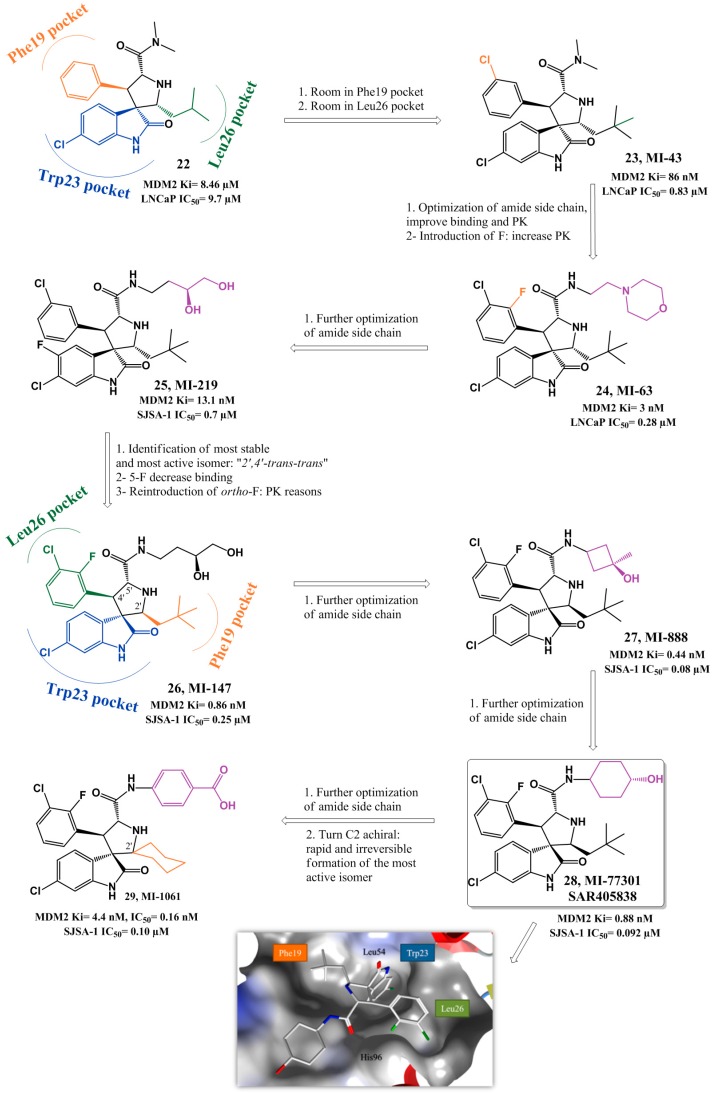
Spiropyrrolidine scaffold optimization. Docking pose of compound **28** in MDM2 (PDB 3LBL). MDM2 surface is colored in blue for hydrophilic areas and grey for hydrophobic areas. Compound **28** is depicted in stick model and is colored according to element type: white for carbon atoms, blue for nitrogen atoms, red for oxygen atoms, bright green for fluorine, and dark green for chlorine atoms.

Taking in consideration that p53 Trp23 side chain (indole group) seems to be critical for p53-MDM2 interaction, by burying deep inside p53 hydrophobic pocket and establishing a hydrogen bond (NH) to the MDM2 backbone (carbonyl), the oxindole moiety was believed to perfectly mimic this residue. Moreover since many natural anticancer products, such as spirotryprostatin A and alstonisine, have a spirooxindole main core, it was rationalized and later corroborated by *in silico* studies that the spiropyrrolidine ring could provide the necessary rigidity to the spirooxindole scaffold, from which two extra hydrophobic groups could be projected in the required orientation to mimic the other two residues of p53. From this structure-based design an initial lead compound arose in 2005 (**22**, Figure 8) with a FP *K*i of 8.46 µM (Wang group, University of Michigan). Computational modeling suggested that optimization could be achieved by varying the isobutyl substituent and by introducing small substituents in the *meta*-position of the phenyl ring (room in Leu26*_(p53)_* and Phe19*_(p53)_* pocket respectively still available). Therefore, structure-activity relationship (SAR) studies led to MI-43 (**23**, FP *K*i = 86 nM, WST-8 LNCaP IC_50_ = 0.83 µM). This compound also showed good selectivity over normal cells and cancer cells with deleted p53 [90].

Further *in silico* investigation into the interaction of compound **23** and p53 with MDM2, suggested that a possible fourth residue (Leu22) could be mimicked. The Leu22*_(p53)_* pocket is partially exposed to solvent and therefore mimicking this residue could result not only in an increase of potency but also in PK improvement, since it could allow the presence of polar groups. A second round of SAR studies was devised, having mainly this observation in consideration, leading to MI-63 (**24**) with a 2-morpholin-4-yl-ethylamine group. Docking studies revealed that not only this side chain could mimic Leu22 but the morpholine oxygen could possibly interact by H bonding with Lys90 in MDM2 (mimicking p53 Glu17). Furthermore the introduction of a fluorine atom in the phenyl group, a frequently effective strategy to increase metabolic stability, augmented the potency (FP *K*i = 3 nM, WST-8 LNCaP IC_50_ = 0.28 µM) [91,92]. However, due to the fact that compound **24** had only a modest oral bioavailability, additional investigations, especially on the polar morpholinyl substituent were performed. It was found that changing to a butyl-1,2-diol significantly improved PK (MI-219: **25**, FP *K*i = 13.1 nM, WST-8 SJSA-1 IC_50_ = 0.7 µM and MI-147: **26**, FP *K*i = 0.6 nM, WST-8 SJSA-1 IC_50_ = 0.2 µM) [93]. Nevertheless these new derivatives still required high oral doses (200–300 mg/kg) to achieve tumor growth inhibition in mice xenograft models, and most important a complete tumor regression remained elusive [94]. More recently this last goal was attained with MI-888 (**27**, FP *K*i = 0.44 nM, WST-8 SJSA-1 IC_50_ = 0.08 µM) [95] and MI-77301/SAR405838 (**28**, FP *K*i = 0.88 nM, WST-8 SJSA-1 IC_50_ = 0.092 µM) [96]. These compounds were synthesized in a new series of optimizations that continued to focus on the 5′ pyrrolidine tail chain, especially by introducing conformational constrain, while attempting to increase metabolic stability [95].

Recently it was discovered that some of these spiropyrrolidine oxindoles can suffer reversible ring-opening and cyclization of the pyrrolidine ring in protic solution, giving rise to different stereoisomers with different binding affinities to MDM2 [97]. Therefore, in 2015, a second generation of spirooxindoles emerged that possess symmetrical substituents at C2′ position of the pyrrolidine ring that allow a rapid and irreversible conversion to the most active diastereoisomer (MI-1061: **29**, FP *K*i = 0.16 nM, WST-8 SJSA-1 IC_50_ = 0.10 µM) [98]. Compounds **27** and **28** from the first generation were already synthesized having in consideration the desired stereochemistry. Interestingly the best diastereomer revealed a different and better binding to MDM2 with the neopentyl and phenyl ring occupying now Phe19*_(p53)_* and Leu26*_(p53)_* pockets respectively (Figure 8, represented for compound **26**). Furthermore their side chain carbonyl is capable of establishing a H bond with the imidazole side chain of His96 and the terminal hydroxyl group with the Lys94 side chain [96].

Compound **28** advanced into clinical trials in 2012 sponsored by Sanofi. It displays more than 100-fold selectivity over cell lines with mutated or deleted p53, activating a p53-dependent pathway leading to cell-cycle arrest and/or apoptosis in cancer cells *in vitro* and *in vivo* xenograft tumor models. A complete tumor regression was achieved at 100 mg/kg with a daily dose for 9 days and at 200 mg/kg with a single oral dose in SJSA-1 mice xenograft [96].

**Figure 9:**
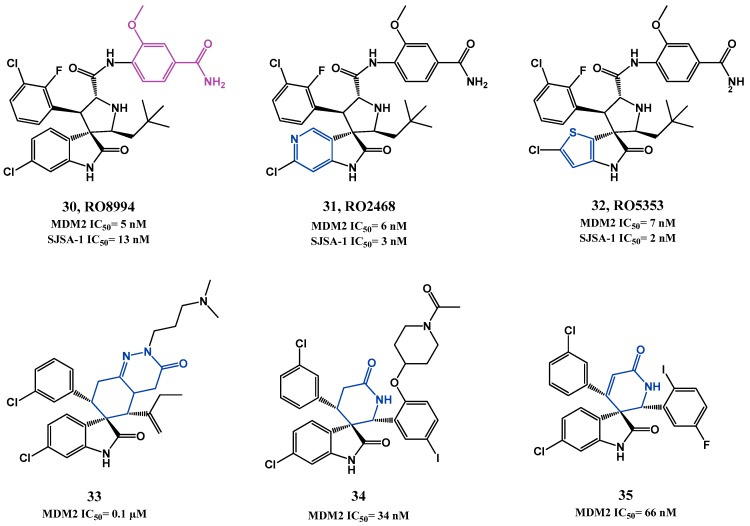
Spiropyrrolidines and others spiro-heterocyclic-oxindole derivatives.

In 2014, Hoffmann-La Roche published two other papers describing further optimizations of spiro[oxindole-3,3′-pyrrolidines], having in consideration the beneficial PK and potency improvement obtained when a phenyl derivative group is attached to the amide side chain. RO8994 (**30**, HTRF IC_50_ = 5 nM, SJSA-1 IC_50_ = 13 nM, Figure 9) emerged in a SAR study focused especially in additional modifications to this side chain [99,100]. Bioisosteric substitution of the 6-chlorooxindole moiety led to compounds RO2468 (**31**, HTRF IC_50_ = 6 nM, MTT SJSA-1 IC_50_ = 3 nM), and RO5353 (**32**, HTRF IC_50_ = 7 nM, MTT SJSA-1 IC_50_ = 2 nM). Although the *in vitro* and *in vivo* activities were comparable to those of RO8994, these compounds showed improved selectivity between wt p53 cell lines and mut p53 cell lines [101].

Several patents have emerged from Hoffmann-La Roche during the last 10 years covering spiro[oxindole-3,3′-pyrrolidines] and other spiro-heterocyclic-oxindole based compounds (e.g., **33**, **34** and **35**) that inhibit the p53-MDM2 interaction [39,42,65].

**Figure 10:**
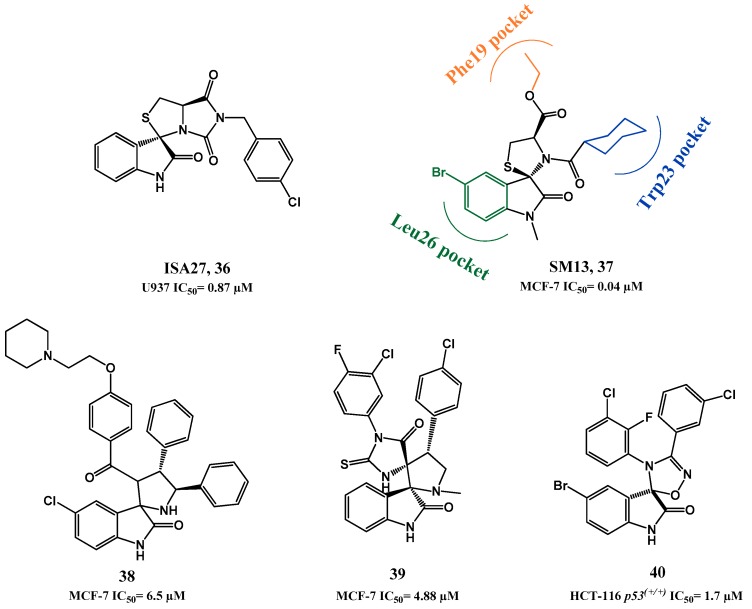
Spiropyrrolidine, spirothiazolidine, and spirooxadiazoline oxindole derivatives with anti-cancer activity.

In 2010, Gomez-Monterrey *et al.* synthesized a series of spiro[oxindole-3,3′-thiazolidines], with ISA27 emerging as the most potent derivative in cell lines (**36**, MTT U937 IC_50_ = 0.87 µM, Figure 10). Destabilization of p53-MDM2 interaction by compound **36** was established first by NMR analysis (AIDA method) [102], and later complemented by an in depth *in vitro* validation in human glioblastoma multiforme [103].

A second round of SAR studies was focused on opening the ISA27 imidazole ring in an attempt to increase structural diversity and introduce extra potential binding points. This was anticipated to allow a better fitting into the p53 pocket, since ISA27 most likely only mimics two of the three pivotal p53 amino acids [104]. SM13 (**37**, MTT MCF-7 IC_50_ = 0.04 µM) emerged from this study. Docking studies suggested that the 3-cyclohexylcarboxy moiety occupies the Trp23*_(p53)_* pocket, and the ethyl ester side chain the Phe19*_(p53)_* pocket. The Leu26*_(p53)_* pocket is only slightly occupied by the oxindole ring, but the authors suggested that this drawback seems to be somewhat compensated by extra hydrophobic interactions gained through the *N*1-methyl group. *In vitro* inhibition of p53-MDM2 interaction was evaluated for both compounds using an ELISA binding assay. At 5 µM ISA27 (**36**) and SM13 (**37**) inhibited 25% and 30% of the interaction respectively (nutlin-3 inhibited 19%). However, both compounds were also effective in cancer cell lines with mutated p53. A detailed study to clarify the mechanism of action of SM13 suggested that besides inhibiting p53-MDM2 interaction, this compound acts in a manner strictly dependent on p53. No apoptosis induction was observed in FRO cells (null p53) and only activation of p53-dependent mitochondrial apoptotic pathway was observed in Kat-4 (mut p53) due to its lack of p53 transcriptional activity [105].

More recently, Kumar *et al.* reported spiro[oxindole-3,2′-pyrrolidines] that seemed to modulate p53 *in vitro* and *in vivo* [106]. However, the compounds did not show selectivity between breast cancer cell lines with wt p53 (MCF-7) and mut p53 (MDA-MB-231), and although an increase in MDM2 levels was observed, no studies were focused in the p53-MDM2 interaction (**38**, MTT MCF-7 IC_50_ = 6.5 µM, Figure 10). Also, Ivanenkov *et al.* reported dispiro compounds with a spiropyrrolidine oxindole moiety that can potentially interfere with p53-MDM2 interaction by *in silico* comparison with known MDM2 antagonists (**39**, MTT MCF-7 IC_50_ = 4.88 µM) [107]. Our research group has also recently developed a family of spiroisoxazoline oxindoles, structural analogues of spirooxindole pyrrolidines, in order to identify new MDM2 inhibitors. The compounds were shown to induce cell death by apoptosis and to inhibit the p53-MDM2 interaction in a cell-based assay [108,109]. Following this work, we synthesized a family of spirooxadiazoline oxindoles in which the spiroisoxazoline carbon C-4′, with tetrahedral molecular geometry, is substituted by a nitrogen atom. Nine compounds showed an antiproliferative activity in cell lines below 10 µM, and four compounds were more active than the positive control nutlin-3a in HCT 116 p53^(+/+)^ cell line. Moreover, they were shown to induce p53 stabilization and transactivation, to induce apoptosis, and to inhibit the interaction between p53 and MDM2 in a live-cell bimolecular fluorescence complementation assay. Compound **40** was one of the most potent compounds in the HCT 116 p53^(+/+)^ cell line (MTS HCT-116 p53^(+/+)^ IC_50_ = 1.7 µM, Figure 10) [110]. Furthermore, we developed a library of spiropyrazoline oxindoles, containing a five membered ring (pyrazoline) with one more aromatic substituent (the oxygen atom in the isoxazoline ring was replaced by a *N*-Ar group) to develop more potent anti-cancer agents. The compounds were evaluated against the MCF7 breast cancer cell line. The most active compounds had activities around 7 µM, and were selective over MDA-MB-231 tumor cells and non-cytotoxic against Hek 293T non-tumor cells [111,112].

**Figure 11:**
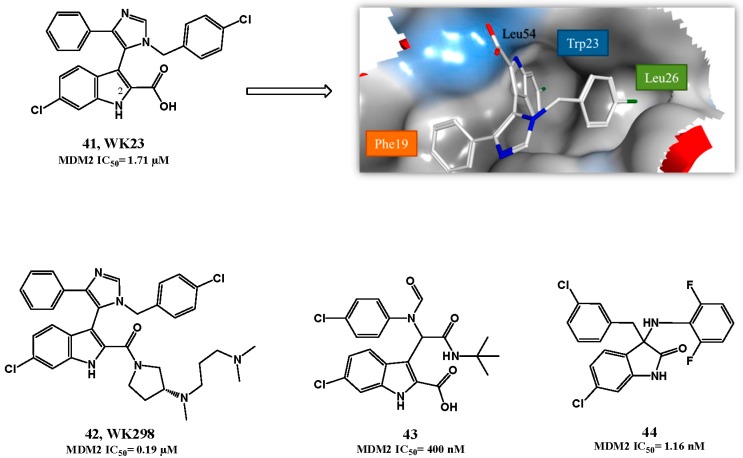
Indolyl derivatives. Right upper quadrant: structure of compound **41** bound to MDM2 (PDB 1YCR). MDM2 surface is colored in blue for hydrophilic areas and grey for hydrophobic areas. Compound **56** is depicted in stick model and is colored according to element type: white for carbon atoms, blue for nitrogen atoms, red for oxygen atoms, and green for chlorine atoms.

Compounds detaining an imidazole-indole scaffold were simultaneously and independently developed by Novartis and the University of Pittsburgh [113,114]. Compounds WK23 (**41**, FP IC_50_ = 1.71 μM, Figure 11) and WK298 (**42**, FP IC_50_ = 0.19 μM) emerged as p53-MDM2 interaction inhibitors. The two molecules only differ by the substituent attached to position 2 of the 6-chloroindole moiety and consequently the central core that mimics p53 is the same. As already observed for other MDM2 inhibitors, a co-crystal structure reveal that the 6-chloroindole ring mimics the p53 Trp23 with the 6-chloro substituent enhancing the interaction by penetrating deeply in the pocket. Moreover, the indole nitrogen atom forms a hydrogen bond with Leu54. The *para*-chlorobenzyl group fills the Leu26 pocket and the phenyl group interacts with the Phe19*_(p53)_* pocket. The additional tail in **42** enhances not only PK properties, but also protects the Phe19*_(p53)_* pocket from solvent [114].

The recognition that an indole/oxindole moiety function was an excellent Trp23 mimetic moiety gave rise to several other potential good compounds (e.g., **43**, FP *K*i= 400 nM and **44**, HTRF IC_50_ = 1.16 nM) [65,115].

**Figure 12:**
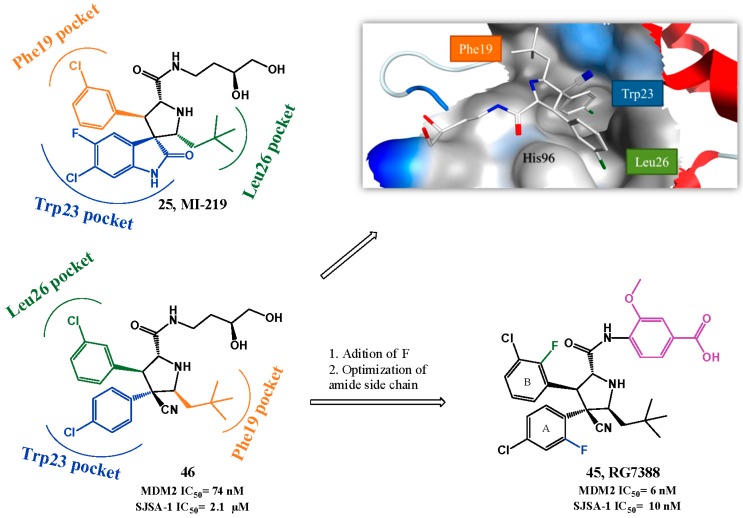
Pyrrolidine-2-carboxamide scaffold optimization. Right upper quadrant: crystal structure of compound **46** bound to MDM2 (PDB 4JRG). MDM2 surface is colored in blue for hydrophilic areas and grey for hydrophobic areas. Compound **46** is depicted in stick model and is colored according to element type: white for carbon atoms, blue for nitrogen atoms, red for oxygen atoms, and green for chlorine atoms.

RG7388 from Hoffman-La Roche (**45**, Figure 12) was designed based on the spiropyrrolidine oxindole MI-219 structure **25** and considering that an aromatic moiety would be better to mimic the Leu26 of p53. The presence of a nitrile group would favor the necessary “*trans-trans*” configuration (between rings A and B, and ring A and neopentyl group, Figure 12) required for better interaction with MDM2. Optimization to RG7388 was mainly focused on the amide side chain of compound **46** (HTRF IC_50_ = 74 nM, MTT SJSA-1 IC_50_ = 2.1 µM). Compound **45**, with a methoxy *para*-benzoic acid moiety, was the most potent derivative with the best PK properties (**45**, HTRF IC_50_ = 6 nM, MTT SJSA-1 IC_50_ = 10 nM). Furthermore, the addition of fluorine to both phenyl rings also contributed to increase binding to MDM2. RG7388 exhibited more than 100-fold selectivity over cell lines with mutated p53, activated the p53 pathway, promoted tumor regression at 25 mg/kg with daily doses in SJSA-1 mice xenograft [116,117] and is currently in clinical trials.

**Figure 13:**
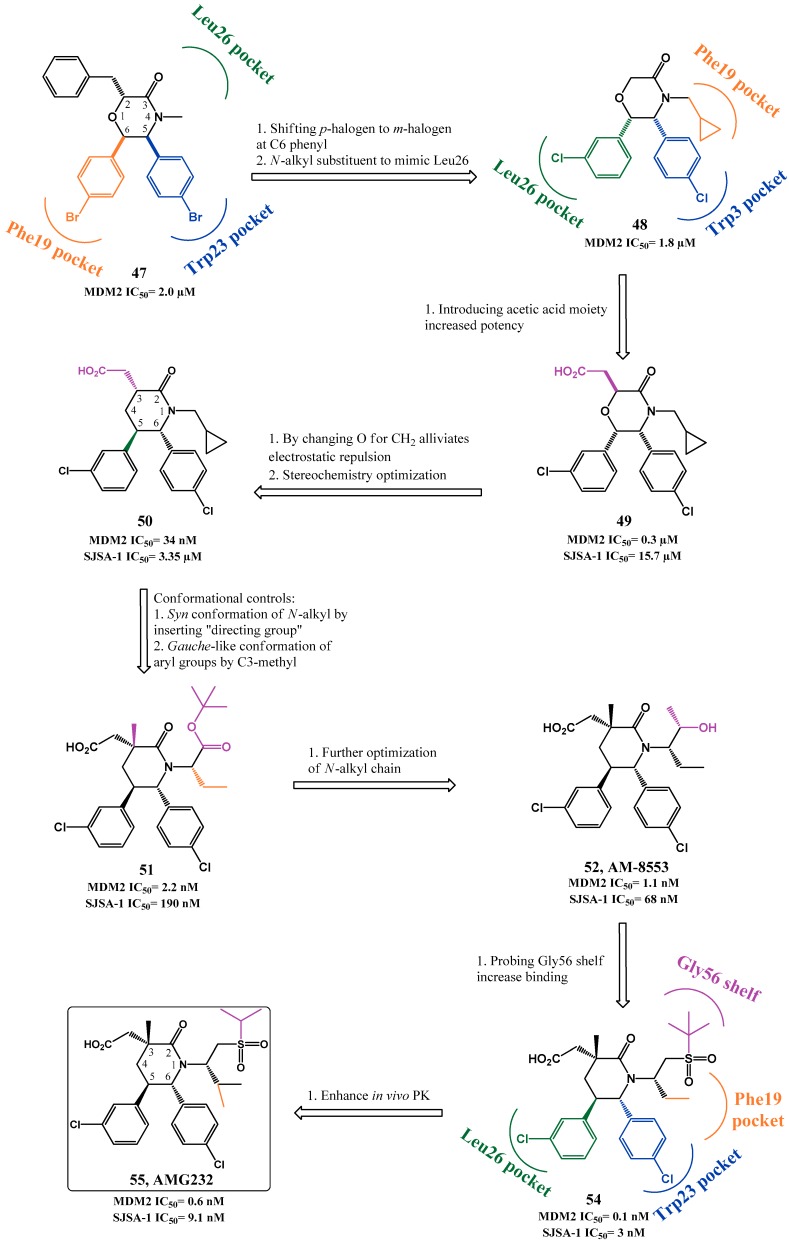
Piperidinone scaffold optimization to AMG232.

In 2012, morpholinones were described by Amgen as p53-MDM2 interaction inhibitors (**47**, HTRF IC_50_ = 2.0 µM, Figure 13) [118,119]. A co-crystal structure of **47** with MDM2 showed that the 6- and 5-*para*-bromophenyl rings occupy Phe19*_(p53)_* and Trp23*_(p53)_* pockets, respectively. Unfortunately the benzyl group was not projected into the Leu26*_(p53)_* pocket and instead it interacted with the Phe55 residue in a shallow hydrophobic shelf region. In an attempt to mimic the Leu26 residue, the *para*-halogen was replaced by a *meta*-halogen on the C6 phenyl ring, leading to a 180° rotation of the morpholinone in the p53 pocket [59,96]. A proper *N*-alkyl substituent would fill the Phe19 pocket (**48**, HTRF IC_50_ = 1.8 µM). An additional SAR study at the C2 position revealed that an acetic acid moiety increased potency by establishing an electrostatic interaction with the His96 residue of MDM2 (**49**, HTRF IC_50_ = 0.3 µM, EdU SJSA-1 IC_50_ = 15.7 µM). However due to the fact that the proximity of this carboxylic acid to morpholinone oxygen could possibly generate electrostatic repulsion and consequently destabilize the right binding conformation, the latter was substituted with a methylene group. This alteration created more potent derivatives, involving now a piperidinone core. Studies of the stereochemical configuration revealed that stereoisomer **50** was the most potent derivative (HTRF IC_50_ = 34 nM, EdU SJSA-1 IC_50_ = 3.35 µM) [118,120].

The next optimizations focused primarily in conformational controls to make sure that the best conformation was favored. In the best binding pose, the *N*-substituent needs to adopt a *syn* (“downward”) orientation in regards to the *para*-chlorophenyl group to properly occupy the Phe19*_(p53)_* pocket. However, since the alternative *anti* conformation is more stable, it was envisaged that introducing a “directing” group at the α-carbon to the piperidinone nitrogen could properly direct the desired group into the pocket. Another important orientation aspect observed in the binding conformation is the necessity of C5 and C6 aryl groups to adopt a *gauche*-like orientation instead of the more stable *anti*-like orientation. For that, introducing another substituent at C3 position could create a 1,3-steric repulsion to the *anti*-like orientation, favoring the desired one (**51**, HTRF IC_50_ = 2.2 nM, EdU SJSA-1 IC_50_ = 190 nM) [119,121].

**Figure 14:**
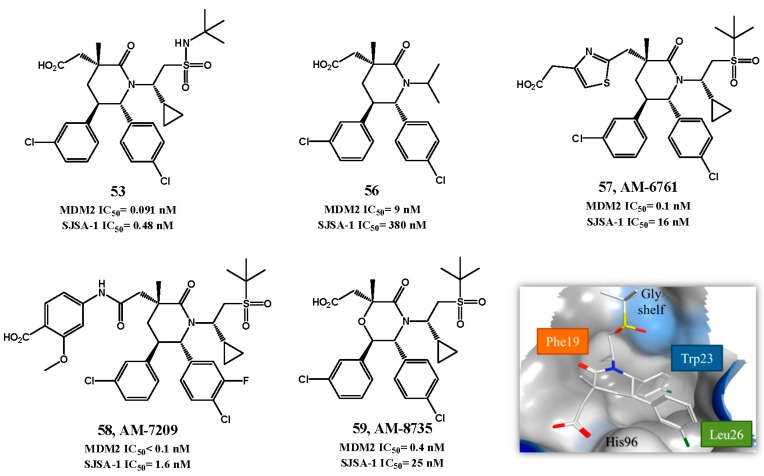
Piperidinone and morpholinone derivatives. Right lower quadrant: crystal structure of compound **54** bound to MDM2 (PDB 4OAS). MDM2 surface is colored in blue for hydrophilic areas and grey for hydrophobic areas. Compound **54** is depicted in stick model and is colored according to element type: white for carbon atoms, blue for the nitrogen atom, red for oxygen atoms, yellow for the sulfur atom, and green for chlorine atoms.

In an attempt to improve the PK properties further optimizations were performed in the *N*-alkyl chain, leading to AM-8553 (**52**, HTRF IC_50_ = 1.1 nM, EdU SJSA-1 IC_50_ = 68 nM) [119,122]. This compound was able to inhibit tumor growth, but only promoted partial tumor regression in a SJSA-1 xenograft tumor model [123]. Therefore, further optimization was pursued. The co-crystal structure of **52** with MDM2 showed a shallow hydrophobic cleft near Phe19*_(p53)_* pocket that could be filled in order to enhance binding. Therefore, several derivatives were synthesized containing different *N*-side chains [124], leading to the very potent sulfonamide piperidone **53** (HTRF IC_50_ = 0.091 nM, EdU SJSA-1 IC_50_ = 0.48 nM, Figure 14) [125]. However, the sulfonamides proved less metabolically stable than **52**. Further optimizations led to compound **54** (HTRF IC_50_ = 0.1 nM, EdU SJSA-1 IC_50_ = 3 nM, Figure 13) and compound AMG232 (**55**, HTRF IC_50_ = 0.6 nM, EdU SJSA-1 IC_50_ = 9.1 nM) [124]. Nevertheless it is noteworthy to point out that even a simple *N*-group can give rise to potent derivatives (**56**, HTRF IC_50_ = 9 nM, EdU SJSA-1 IC_50_ = 0.38 µM, Figure 14) [126]. Compound **55** entered clinical trials in 2012. Although compound **54** was more active than **55** it offered poorer PK properties in *in vivo* studies.

Binding of **55** with MDM2 was extrapolated from the co-crystal structure of **54** with MDM2 (Figure 14). As expected the three pivotal p53 amino acids Phe19, Trp23 and Leu26 are being mirrored by the isopropyl, C6 aryl group and C5 aryl group, respectively. Two substituents interact with His96: the C5 aryl engages in a π-π stacking, while the carboxylate function forms a hydrogen bond with its imidazole side chain. In addition, the sulfone group projects the terminal isopropyl into the glycine shelf region. Compound **55** was able to induce complete tumor regression in 10 out of 12 SJSA-1 xenograft mice (60 mg/Kg administered orally once daily) [124,127]. Additional SAR studies were performed mainly by searching for new replacements for the carboxylate moiety that still allowed the interaction with His96 and possibly enhanced potency. This led to AM-6761 (**57**, HTRF IC_50_ = 0.1 nM, EdU SJSA-1 IC_50_ = 16 nM) with potency similar to **55**. Interestingly these two derivatives have different metabolic profile that can be of interest to explore. Compound **57** is primarily metabolized by oxidative pathways, while compound **55** is metabolized mainly by glucuronidation of the carboxylate moiety [128]. Further optimization led to AM-7209 (**58**, HTRF IC_50_ < 0.1 nM, EdU SJSA-1 IC_50_ = 1.6 nM) [129].

**Figure 15:**
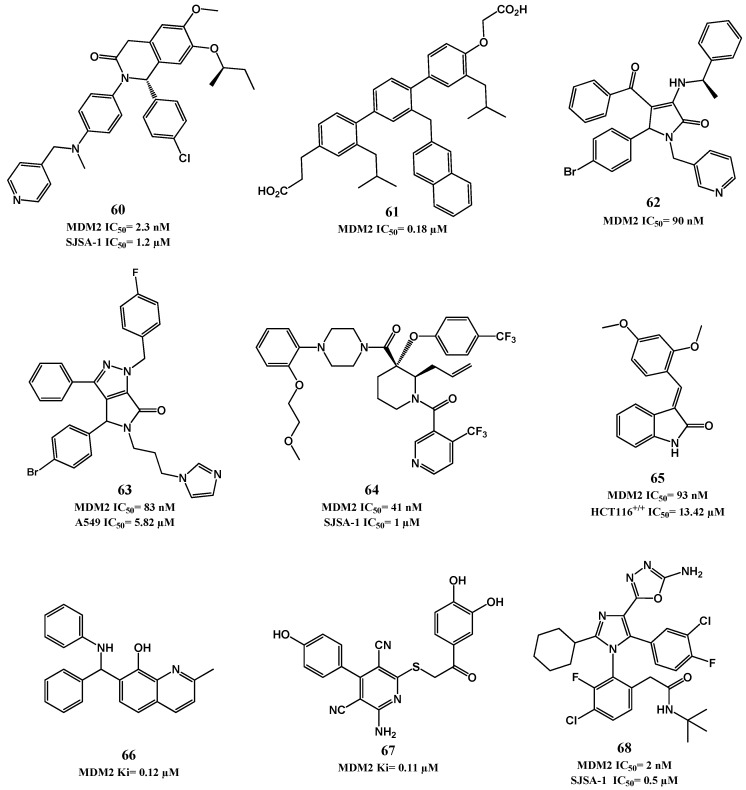
p53-MDM2 interaction inhibitors.

Alongside the synthesis of piperidones, Amgen continued to optimize morpholinone derivatives, leading to AM-8735 (**59**, HTRF IC_50_ = 0.4 nM, EdU SJSA-1 IC_50_ = 25 nM) [130]. In Table 2 and Figure 15 are described other p53-MDM2 interaction inhibitors with activity in the nanomolar range for MDM2 that have been reported throughout the years.

Apart from RG7112, MI77301, RG7388, and AMG232, another four small molecules have advanced into clinical trials, but no structural information is available: MK-8242, RO6839921, CGM097 and DS-3032b developed by Merck, Hoffmann-La Roche, Novartis International and Daiichi Sankyo, respectively [50].

### 2.2. MDMX and Dual MDM2/MDMX Inhibitors

Although MDM2 is the foremost negative regulator of p53, MDMX has been recognized more recently as a critical discrete p53 modulator, and in fact its overexpression is observed in several cancers [145,146]. MDMX is structurally related to MDM2, but lacks its p53 ubiquitin-mediated degradation signal. However it is able to control p53 activity mostly by inhibiting the p53 transcription function. In addition, MDMX protein levels are regulated by MDM2 ubiquitin-mediated degradation [147]. Since inhibiting p53-MDM2 interaction increase MDM2 levels by autoregulatory feedback loop, and therefore can facilitate MDMX degradation, the problem of MDMX presence could be lessened when targeting this interaction. However, although reduced MDMX levels are observed in many cancers after treatment with nutlin-3a, the effectiveness of the inhibitor can still be compromised, especially in tumors overexpressing MDMX [148].

**Figure 16:**
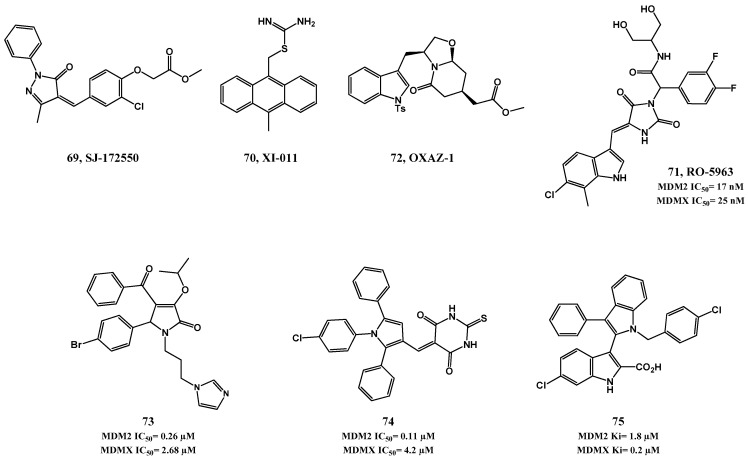
MDMX and dual MDM2/MDMX inhibitors.

The first small molecule inhibitor of MDMX (SJ-172550, **69**, Figure 16) was only reported in 2010. This compound was found to bind reversibly to MDMX in the p53 binding pocket, and showed cytotoxicity in MDMX-amplified retinoblastoma cell line Weri1 [149]. Further investigation revealed that compound **69**, through reversible covalent binding, seemingly locks MDMX into a conformation that is unable to bind p53. This complex mechanism of action was revealed to be dependent on several factors, limiting this compound as a feasible lead compound [150].

Compounds XI-006 (NSC207895) and XI-011 (NSC146109, **70**) were identified in a HTS assay as activators of p53-dependent transcription [151]. The mechanism of action of these compounds was unveiled in 2011 to involve inhibition of MDMX expression, by repressing *MDMX* promoter and subsequent down-regulation of its mRNA [28,152]. Recently it was also suggested that XI-011 was capable of disrupting the p53-MDMX interaction [153].

Although initially some reports demonstrated the beneficial aspect of inhibiting MDMX alone, specially due to its lower toxicity to normal tissues [148], it is now recognized that a full p53 activation outcome is favored and more likely to be achieved with dual inhibition of MDM2 and MDMX. In fact, compounds possessing an imidazo-indole scaffold act as dual inhibitors (e.g., WK298, **42**, MDM2 FP IC_50_ = 0.19 µM; MDMX FP IC_50_ = 19.7 µM, Figure 11). The co-crystal structure of WK298 with MDMX confirmed that the main aspects that need to be addressed for an adequate inhibition of both proteins lies in the three subpockets Phe19*_(p53)_*, Trp23*_(p53)_* and Leu26*_(p53)_*. The difficulty of dual inhibition seems to be attributed mainly to Leu26*_(p53)_* pocket, which is quite different in the two proteins, and may be the reason for a much weaker binding observed for most of the known MDM2 inhibitors. From this observation it can be assumed that the common feature of possessing a chlorophenyl group, although ideal for MDM2, is not optimal for mimicking p53 Leu26 interaction with MDMX [114].

More recently, indolyl-hydantoin derivatives were reported to potently block p53 binding with both MDM2 and MDMX. Specifically, compound RO-5963 (**71**, MDM2 TR-FRET IC_50_ = 17 nM; MDMX TR-FRET IC_50_ = 25 nM) showed p53-MDM2 inhibitory activity similar to that of nutlin-3a and approximately 400-fold better p53-MDMX inhibitory activity than nutlin-3a [154]. Other small molecules (Figure 16) have been identified as dual inhibitors in the last years, including tryptophanol-derived oxazolopiperidone lactam **72** [155], pyrrolidones (**73**, MDM2 FP IC_50_ = 0.26 µM; MDMX FP IC_50_ = 2.68 µM) [134], triaryl-pyrroles (**74**, MDM2 FP IC_50_ = 0.11 µM; MDMX FP IC_50_ = 4.2 µM) [156], and 2,3′-bis(1′H-indoles) (**75**, MDM2 FP *K*i = 1.8 µM; MDMX FP *K*i = 0.2 µM) [157].

### 2.3. p53 Reactivators: wt p53 and Mut p53 Targeting Small Molecules

Apart from inhibition of p53-MDMs interactions, other approaches have been used in order to reactivate wild-type p53 including targeting MDM2 E3 ligase, sirtuins, the calcium-binding protein S100B, *etc*. In addition, p53 function perturbations are also observed in cancers as a result of point mutations to the *TP53* gene, representing the most frequent mutated gene observed in human cancers. Although in some cases such events can lead to loss of p53 protein expression, as a result of frameshift or nonsense mutations, more frequently it leads to a single amino acid substitution in the protein (missense mutations). These replacements take place more frequently within the DNA binding domain leading to loss or attenuation of wild-type p53 function. p53 mutants can generically be categorized into two groups: *contact mutants*, in which the amino acid replacement affects p53’s ability to bind to DNA without significantly affecting its conformation; and *conformational mutants*, in which the substitution promotes a disruption of the normal p53 tertiary structure. However, the signaling outcome cannot be oversimplified in two possible outcomes, since different mutations can lead to different degrees of inhibition/loss of p53 function, as well as to the acquisition of increased function. This gain of function can further promote tumorigenesis, potentially leading to a more aggressive cancer profile. Gain of function by mutant p53 can be a consequence of the activation of other signaling pathways, through interaction with other proteins, such as the p53 family members p63 and p73, and indirectly affecting gene expression. The intricacy of this mutant p53 signaling disturbance effect is further amplified by the fact that different expression of its targets can be met in different tissues, potentially generating the variety of cancer phenotypes observed, even for the same point mutation [158,159].

A variety of small-molecule therapy strategies can be devised when dealing with mutant p53, such as: (i) reactivation of wild-type p53 function; (ii) interference with the interaction between mutant p53 and other proteins; (iii) promotion of mutant p53 degradation; and (iv) downstream interference in the mutant p53 signaling pathways.

**Figure 17:**
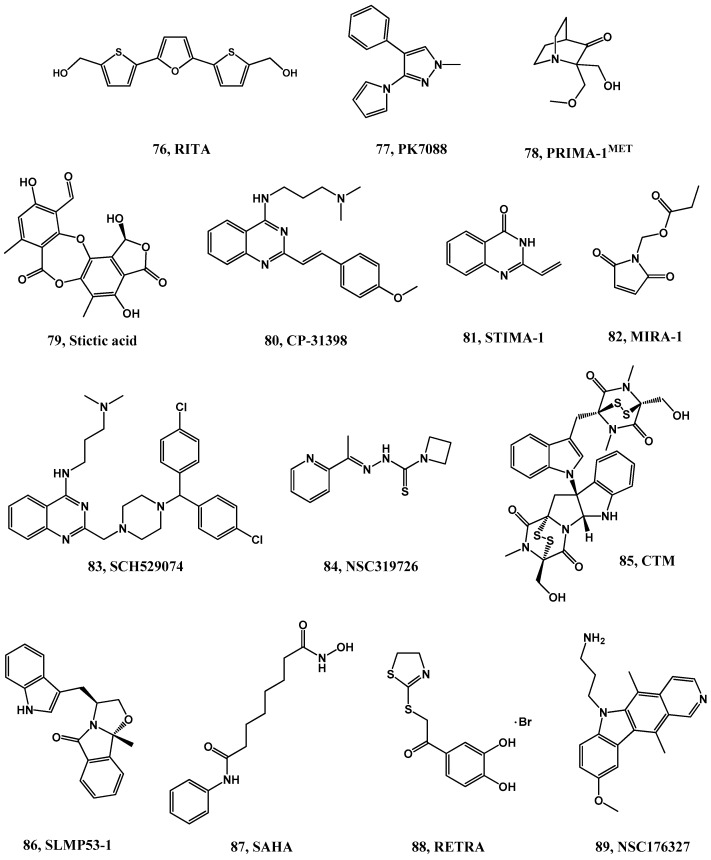
Compounds targeting mutant p53.

To date, there are some small molecules reported to target mut p53. For most of them, the mechanism of action is still not fully understood. Moreover, some of these molecules show p53-independent activity and cytotoxicity in cancers with wild-type p53 [21,158,159,160]. For example, Reactivation of p53 and Induction of Tumor Cell Apoptosis (RITA, **76**, Figure 17) was identified to suppress selectively HCT116 cell line expressing wild-type p53 over the p53 null counterpart cell line. It was proposed that RITA binds to the p53 *N*-terminal domain, inducing a conformational change that prevents its binding to MDM2, thus restoring p53-transcriptional activity [161,162]. Interestingly, more recently RITA’s mechanism of action was extended to cell lines presenting mutated p53. Presumably the binding to mutated p53 might affect the core domain folding in a way that potentially restore its DNA binding ability [163]. This dual targeting increases the application scope of RITA turning it into a very promising lead compound to rescue p53 regardless of the nature of its inactivation [164]. In addition, RITA can promote down-regulation of MDMX selectively in wild-type cancer cells through a pathway independent of MDM2 [165]. Novel analogues slightly more active and selective have been already synthesized [166,167].

Several small molecules were reported to restore wt p53 function. Specifically, PhiKan083 [168], PhiKan5196 [169], and PK7088 (**77**) [170] are small molecules that target the Y220C p53 mutation, a mutation that creates a druggable surface crevice that destabilizes the protein.

PRIMA-1, and PRIMA^MET^ (APR-246, **78**) [171,172], target the R175H and R273H p53 mutations. These molecules are converted into compounds capable of forming adducts with mutant p53 cysteine residues. APR-246 is currently in clinical trials [173]. Another small molecule that acts on mutated p53 is stictic acid (**79**) that in human osteosarcoma cells, exhibits dose-dependent reactivation of p21 expression for mutant R175H more strongly than does PRIMA-1 [174].

CP-31398 (**80**), a styrylquinazoline, emerged from a high throughput screen for compounds that restore a wild-type-associated epitope (monoclonal antibody 1620) on the DNA-binding domain of the p53 protein. CP-31398 (**80**) stabilizes exogenous p53 in p53 mutant (mutant p53 V173A and R249S), wild-type, p53-null human cells, and in MDM2-null p53^−/−^ mouse embryonic fibroblasts [175,176].

STIMA-1 (a compound with some structural similarities to CP-31398) and MIRA-1, identified in a cellular screening, are also reactivators of mutant p53. Both STIMA-1 (**81**) [177] and MIRA-1 (**82**) [178], probably restore the wt p53 function by reacting with thiols and amino groups in p53. MIRA-1 reactivates mutant p53 R175H and R273H, but it was recently reported to have off-target effects [160].

SCH529074 (**83**) was identified using a screen based on a p53 DNA binding assay. SCH529074 (**83**) restores DNA binding activity to two mutant forms of p53, the contact point mutant R273H and the structural mutant R249S. Moreover, it binds to p53 core domain and it is believed to act as a chaperone, not binding covalently to p53. It also inhibits MDM2-mediated ubiquitination [179]. NSC319726 (ZMC1, **84**) is a reactivator of R175H mutant p53 and functions as zinc metallochaperone, providing an optimal concentration of zinc to p53, promoting proper folding of p53-R175H [180,181].

The natural compound, chemotin (CTM, **85**), was identified to act as a mut p53 reactivator using a cell-based, high-throughput small-molecule screen. CTM inhibits growth of cancer cells harboring mutant p53 R175H *in vitro* and *in vivo*, binds to Hsp40 and increases the binding capacity of Hsp40 to the p53 R175H mutant protein, causing a potential conformational change to a wild-type-like p53 [182]. More recently, the enantiopure tryptophanol-derived oxazoloisoindolinone (SLMP-53-1, **86**) was identified as a novel reactivator of wild-type (wt) and mut p53. SLMP-53-1 enhanced p53 transcriptional activity, restored wt-like DNA binding ability to mut p53R280K, and showed promising p53-dependent synergistic effects with conventional chemotherapeutics. Moreover, in xenograft mice models, it inhibited the growth of wt/mut p53-expressing tumors, but not of p53-null tumors, without apparent toxicity [183].

Vorinostat (SAHA, **87**) was described as a histone deacetylase inhibitor and can destabilize the complex formed between HSP90 and mut p53. This complex inhibits E3 ubiquitin ligases MDM2 and CHIP, causing mut p53 stabilization. Moreover, the IC_50_ values were profoundly lower in mut p53 cancer cells (T47D IC_50_ = 1.7 µM; MDA231 IC_50_ = 1.1 µM; ES2 IC_50_ = 1.9 µM) over wt p53 cancer cells (RKO IC_50_ = 393.0 µM; HCT116 p53^(+/+)^ IC_50_ = 128.0 µM) [184]. Vorinostat is already being used in the clinic for treatment of cutaneous T cell lymphoma [173].

Other small molecules that act on mutated p53 are RETRA (**88**) [185] and NSC176327 (**89**) (a derivative of ellipticine) [186] which promote the release of p73 protein from the blocking complex with mutant p53. In particular, RETRA is active against tumor cells expressing a variety of p53 mutants (His-273, Trp-248, Glu-266, Lys-280), while not affecting normal cells [185].

## 3. Concluding Remarks

Due to the unquestionable contribution of p53 to the preservation of genomic integrity, it is not surprising that tumor pathogenesis and development involves some sort of p53 impairment. Hence, restoring p53 function in cancer cells represents a valuable anticancer approach. Several strategies are being developed and in particular targeting p53-MDM2 interaction has emerged as a promising approach, when dealing with cancers that retain wild-type p53 function. In particular, seven p53-MDM2 interaction inhibitors have entered clinical trials. However, it was recently shown by Aziz *et al.* that non-genotoxic p53 activation by the MDM2 inhibitor, nutlin-3a, can lead to the acquisition of somatic mutations in p53 [59]. If these studies are confirmed with other MDM2 inhibitors, they will have implications for the potential clinical use of MDM2 antagonists.

More recently, the strategies to target p53 involve the dual inhibition of the p53-MDM2 and p53-MDMX interactions to effectively activate wild-type p53. In the case of more aggressive cancers where p53 is mutated, the strategy involves the development of small molecules that target mut p53. 

As one of the main problems when dealing with any chemotherapeutic agent is its toxicity to normal cells, it is important to find new drugs that can distinguish cancer cells from normal cells. Non-genotoxic strategies that focus on reactivating p53 can represent a step further in this direction, due to the fact that the outcome of p53 activation relies, among other factors, in the intracellular environment that *per se* is different in cancer and normal cells. In the case of tumors with mutated p53, p53 status represents an inherent difference between these two types of cells and therefore p53-based cyclotherapy can be a very useful strategy. In this approach, first it is given a pretreatment with a low dose of wild-type p53 activating molecule that can trigger a transient cell cycle arrest in normal cells, without affecting cancer cells. Then, by adding a conventional anticancer agent that targets S and M phase, cancer cells will selectively be triggered into an apoptotic outcome [187].

## Figures and Tables

**Table 1:** Cell-free and cell-based *in vitro* assays.

**Cell-Free Binding Assays**	
SPR	Surface plasmon resonance
HTRF	Homogeneous time resolved fluorescence
FP	Fluorescence polarization
NMR-AIDA	NMR-based antagonist induced dissociation assay
ThermoFluor	Thermal denaturation screening assay
TR-FRET	Time-resolved fluorescence energy transfer
ELISA	Enzyme-linked immunosorbent assay
**Cell-Based Assays**	
BrdU	Bromo-2′-deoxyuridine
EdU	5-Ethynyl-2′-deoxyuridine
LCVA	Luminescent cell viability assay
MTT	Tetrazolium salt
SRB	Sulforhodamine B
WST-8	Water soluble tetrazolium salt
**Cell Lines**	
A549	Human lung carcinoma—wild-type p53
Fro	Human anaplastic thyroid carcinoma—null p53
HCT116 *p53*^(+/+)^	Human colorectal cancer—wild-type p53
JAR	Human choriocarcinoma—wild-type p53
Kat-4	Human thyroid tumor—mutant p53
LNCaP	Human prostatic adenocarcinoma—wild-type p53
MCF-7	Human breast adenocarcinoma—wild-type p53
MDA-MB-231	Human breast adenocarcinoma—mutant p53
MHM	Human osteosarcoma—wild-type p53
SJSA-1	Human osteosarcoma—wild-type p53
U-2OS	Human osteosarcoma—wild-type p53
U937	Human lung lymphoblast—wild-type p53

**Table 2:** Other chemical families described for p53-MDM2 interaction inhibitors.

Chemical Family	Compound	MDM2 (IC_50_ or *K*i)	Cell-Based Assay (IC_50_)
Dihydroisoquinolinones [131]	**60**	IC_50_ = 2.3 nM ^a^	1.2 μM (SJSA-1)
Terphenyl derivatives [132,133]	**61**	*K*i = 180 nM ^b^	ND ^f^
Pyrrolidones [134,135]	**62**	IC_50_ = 90 nM ^c^	ND ^f^
Pyrrolo[3,4-*c*]pyrazoles [136,137]	**63**	IC_50_ = 83 nM ^c^	5.8 µM (A549) ^d^
Piperidines [138,139,140]	**64**	IC_50_ = 41 nM ^c^	1.0 µM (SJSA-1) ^d^
3-benzylideneindolin-2-ones [141]	**65**	*K*i = 93 nM ^c^	13.4 µM (HCT116 *p53^(+/+)^*) ^d^
8-hydroxyquinoline [142]	NSC66811 (**66**)	*K*i = 120 nM ^c^	ND ^f^
Pyridine derivative [143]	**67**	*K*i = 110 nM ^c^	ND ^f^
Imidazole [144]	**68**	IC_50_ = 2 nM ^a^	0.5 µM (SJSA-1) ^e^

^a^ TR-FRET; ^b^ ELISA; ^c^ FP; ^d^ MTT; ^e^ YO-PRO^®^-1iodide staining; ^f^ not determined.
